# Acute and Short-Term Effects of Real-Time Feedback Neuromuscular Training on Vertical Jump Performance and Reactive Efficiency in Youth Basketball Players

**DOI:** 10.3390/sports14080362

**Published:** 2026-08-20

**Authors:** Alexandra Reta Iacobini, Pierre Joseph de Hillerin, Vlad Adrian Geantă

**Affiliations:** 1 Doctoral School of Sports Science and Physical Education, Pitești University Center, National University of Science and Technology POLITEHNICA Bucharest, 110040 Pitești, Romania; alexandra.iacobini@spiruharet.ro (A.R.I.); hillerin@live.com (P.J.d.H.); 2 Department of Physical Education, Sport and Kinesitherapy, Faculty of Physical Education and Sport, Spiru Haret University, 030045 Bucharest, Romania; 3 Neuromotrica-Information for Sport and Human Performance Ltd., 021323 Bucharest, Romania; 4 Department of Physical Education and Sport, Faculty of Physical Education and Sport, Aurel Vlaicu University of Arad, 310330 Arad, Romania

**Keywords:** neuromuscular training, real-time feedback, vertical jump, reactive strength, average power, youth basketball, motor control

## Abstract

Real-time feedback-based training has emerged as a potentially time-efficient approach for enhancing neuromuscular performance, yet evidence in youth basketball athletes remains limited. This study examined the acute and short-term effects of a five-day simulator-based neuromuscular training program incorporating real-time feedback on vertical jump performance and reactive efficiency in youth basketball players. A quasi-experimental, single-group repeated-measures design was employed, with performance assessed at three time points: pre-intervention (T1), post-intervention (T2), and 7-day follow-up (T3). Twenty-one male junior athletes (15.1 ± 0.7 years) completed five consecutive sessions on a condition simulation device (ERGOSIM), designed to enhance force control and neuromuscular coordination through real-time visual feedback. Vertical jump performance was assessed using a 15 s repeated jump test (OptoJump), including bilateral and unilateral conditions. Primary outcomes were jump height (H), mean average power (PU), and contact time (CT), analyzed via the MGM-15 method. Repeated-measures ANOVA revealed significant time effects for bilateral performance: H (F(2,40) = 16.593, *p* < 0.001, η^2^_p_ = 0.453), PU (F(2,40) = 19.281, *p* < 0.001, η^2^_p_ = 0.491), and CT (F(2,40) = 6.009, *p* = 0.005, η^2^_p_ = 0.231). From T1 to T3, bilateral jump height increased by 17.0%, accompanied by a 14.5% increase in average power and a 10.3% reduction in contact time. Unilateral performance improved significantly in both limbs, with progressive reduction in inter-limb asymmetry across the intervention period. Performance gains were largely maintained at T3, indicating short-term retention of neuromuscular adaptations. These findings suggest that simulator-based, feedback-driven neuromuscular training represents a time-efficient strategy for optimizing explosive performance and inter-limb symmetry within competitive microcycles although the single-group design and absence of a control group warrant cautious interpretation.

## 1. Introduction

Basketball performance depends on the integrated development of strength, speed, agility, and explosive power, which underpin key game-specific actions such as jumping, sprinting, shooting, rebounding, and rapid changes in direction [1,2,3]. Among these, the ability to generate high levels of force in minimal time, commonly referred to as the rate of force development, is considered a critical determinant of high-level performance [4,5]. Accordingly, optimizing lower-limb power is a primary objective of basketball training because it directly supports acceleration, agility, and vertical jump performance [6,7,8,9].

Vertical jump performance is a key physical attribute in basketball, contributing to both offensive and defensive actions and representing an important target of athletic preparation [1,10,11,12,13]. Among available methods, plyometric training is widely recognized as one of the most effective approaches for enhancing explosive strength and neuromuscular efficiency [6,14,15,16,17,18,19], largely through adaptations in the stretch–shortening cycle, neural activation, and intermuscular coordination [4,20,21,22,23,24,25].

Evidence from experimental studies [10,19,26,27,28,29], meta-analyses [15,21,30] and systematic reviews [7] indicates moderate-to-large improvements in explosive performance, including vertical jump ability, typically ranging between 4% and 15% after 6–12 weeks of training. Similar benefits have also been reported for sprinting and change-of-direction performance in youth and basketball-specific populations [14,23,31]. However, these adaptations usually require several weeks of training and remain influenced by program duration, intensity, volume, and athlete characteristics [32,33].

Although shorter interventions lasting 4–8 weeks have also improved explosive performance [34,35,36,37], they still rely predominantly on mechanical loading strategies that may induce fatigue and prioritize force production over neuromuscular coordination and movement control [38]. In elite youth basketball, in-season competitive microcycles typically span 5–7 days with dense match and practice schedules, leaving limited scope for longer or more disruptive interventions during this period [39]. Within such constraints, there is a practical need to determine whether a compact neuromuscular “booster” microcycle, delivered over approximately five consecutive sessions, can enhance explosive performance without increasing mechanical loading or compromising regular team training.

Emerging technologies may help overcome these limitations by enabling real-time monitoring and modulation of neuromuscular performance [40,41,42]. Sensor-based and feedback-driven systems have shown promising effects on movement quality, motor learning, and performance in youth athletes [43,44,45]. These systems encompass diverse wearable sensors, force platforms, and interactive or game-based interfaces that deliver real-time feedback on movement execution [41,42]. In the present study, we focus on one specific implementation of this broader concept, a simulator-based system that provides force–position feedback during lower-limb extension tasks.

Unlike conventional plyometric approaches, simulator-based systems providing real-time visual feedback may enhance movement control, force regulation and inter-limb coordination through closed-loop neuromuscular control [46,47]. By enabling immediate correction of movement errors, real-time feedback may accelerate motor learning and promote performance improvements within shorter intervention periods [45,48].

However, despite the growing interest in technology-assisted neuromuscular training, most interventions have been evaluated over medium- or long-term periods [49,50]. Consequently, it remains unclear whether significant neuromuscular adaptations can be achieved within a very short intervention window, particularly in youth basketball athletes exposed to dense competitive schedules [51,52]. In addition, inter-limb asymmetries are increasingly recognized as relevant indicators of neuromuscular function and athletic performance in basketball [53,54]. However, there is little evidence on the short-term effects of feedback driven neuro-muscular interventions on inter-limb symmetry [55].

Therefore, the present study aimed to examine the acute and short-term effects of a five-day simulator-based neuromuscular training program incorporating real-time feedback on vertical jump performance, reactive efficiency, and inter-limb symmetry in youth basketball players. Within the constraints of an in-season competitive microcycle, a single-group repeated-measures design was adopted as a pragmatic proof-of-concept approach to test the time-efficiency and short-term retention of feedback-driven neuromuscular training before progressing to larger controlled trials. It was hypothesized that feedback-driven training would accelerate neuromuscular coordination and stretch–shortening cycle optimization, resulting in significant improvements in jump height, average power, and contact time across both bilateral and unilateral conditions within a brief intervention period.

## 2. Materials and Methods

### 2.1. Research Design

A quasi-experimental, single-group pretest–post test design with repeated measures was employed. Performance was assessed at three measurement points: pre-intervention (T1), post-intervention (T2), and follow-up (T3, seven days later). T2 captured the immediate effects of the five-day intervention, whereas T3 assessed short-term retention without additional specialized training. Given the constraints of the in-season competitive period, this design was chosen as a pragmatic first step prior to more controlled comparisons.

### 2.2. Participants

Twenty-one male junior basketball players (age: M = 15.1 ± 0.7 years; height: M = 186.29 ± 7.91 cm; body mass: M = 75.19 ± 12.89 kg) participated in the study. The sample size was determined by the limited availability of these elite athletes during the competitive season. All participants were high-performance athletes competing in the final stages of the National Championship, with several representing the Romanian National Basketball Team. Inclusion criteria required active participation in competitive basketball and familiarity with vertical jump execution. Exclusion criteria ruled out any recent musculoskeletal injuries, lower-limb surgeries, or neurological disorders that could affect performance. Only male athletes were included due to the limited number of eligible female players during recruitment. All participants and their parents or legal guardians were informed about the procedures, risks, and benefits of the study. Written informed consent was obtained from the parents or legal guardians of all athletes, and assent was obtained from the athletes themselves. The study protocol was approved by the institutional ethics committee of Aurel Vlaicu University of Arad, Faculty of Physical Education and Sport (Registration number: 651/06.11.2025), in accordance with the Declaration of Helsinki.

### 2.3. Equipment

#### The ERGOSIM Condition Simulation Device

The intervention utilized the Condition Simulation Device (ERGOSIM Simulator, manufactured by the Romanian National Center for Sport Research, Bucharest, Romania), developed at the same institution between 1973 and 1990 (inventors: Schor, V.; de Hillerin, P.J.; Stupineanu, I.; Feredean, A.; Dinescu, I.). The device was registered as a patented invention under the Romanian State Office for Inventions and Trademarks (OSIM) patent no. 108411/1994.

Over the past decades, ERGOSIM-based psychoneuromotor training protocols have been applied in diverse populations, from preschool children and artistic performers to youth and elite athletes, to develop visual–psychomotor schemes and enhance neuromuscular control under task-specific constraints [56,57,58]. Related methodologies based on the same theoretical framework have also been used to assess neuromuscular control, functional stress responses, and motor coordination in artistic gymnastics and through Cartesian motion analysis [59,60]. In high-performance sport, the ERGOSIM and associated approaches have supported biomechanical analysis, technical error detection, and the management of inter-limb asymmetries in jumping, swimming, and team sport settings [55,61,62,63,64,65]. We acknowledge that most ERGOSIM- and CASINOR-related studies come from a limited group of investigators. Accordingly, we present ERGOSIM here as one specific example of feedback-integrated, sensor-based neuromuscular training.

The ERGOSIM integrates force transducers that record propulsion forces in real time [66]. Its configuration includes an electrodynamic brake system enabling speed-dependent nonlinear resistance control and an electric motor equipped with digital signal processors and position transducers, allowing precise measurement of force and displacement while providing a baseline motion that encourages force production at higher velocities [66].

### 2.4. Neuromuscular Training Protocol

The training program was designed as a short-term, high-feedback intervention aimed at enhancing neuromuscular coordination, rate of force development, and reactive control. The intervention was conducted over five consecutive days, targeting the lower-limb kinetic chain responsible for ankle, knee, and hip triple extension, the biomechanical basis of vertical jumping [4,5]. During the intervention period, athletes maintained their regular basketball training schedule, standardized across the team. This schedule consisted of three on-court team practices and one competitive game within the weekly microcycle, including technical–tactical drills and conditioning elements planned by the club coaching staff. No additional structured strength or plyometric sessions were introduced during the intervention window.

The protocol was based on the CASINOR principle (Computer-Assisted Informational Orthotics), previously described in the Equipment section. Briefly, CASINOR integrates the athlete into a computer-mediated feedback loop by continuously recording force, displacement, velocity, and acceleration while simultaneously providing real-time visual feedback that allows immediate adjustment of force output and movement timing during task execution [67,68,69,70].

This feedback-supported approach is intended to facilitate sensorimotor regulation and rapid movement corrections while progressively reducing reliance on external cues as motor control becomes more refined [71,72]. All exercises were performed under low-resistance, speed-controlled conditions to prioritize neuromuscular control and movement precision rather than maximal force production.

The neuromuscular program comprised three exercises targeting distinct segments of the lower kinetic chain (Table 1). Low loads were applied to prioritize movement control and neuromuscular efficiency. Progression across the five sessions was achieved by increasing average execution speed (AS) and reducing brake intensity from 90% (Tr1–Tr2) to 30% (Tr4–Tr5) (Table 2 and Table 3).

The protocol followed a structured five-day progression (Table 3). Session Tr1 involved alternating unilateral execution under 90% brake intensity, providing slow, controlled movement with sufficient time for motor learning, with rest intervals of 5–6 min between exercises. In Tr2, execution shifted to simultaneous bilateral mode under unchanged brake intensity, requiring enhanced inter-limb coordination while consolidating previously acquired movement mechanics. Tr3 introduced a reduction in brake intensity to 50%, emphasizing faster bilateral movements, with rest intervals extended to 6–7 min to accommodate increased neuromuscular demands. Tr4 further reduced brake intensity to 30%, progressively increasing execution speed and reactive control requirements. Finally, Tr5 introduced a motor-driven baseline speed of 0.5 m/s under 30% brake intensity, establishing a minimum velocity threshold and requiring athletes to generate force above baseline, thereby simulating dynamic high-velocity conditions.

**Figure 1:**
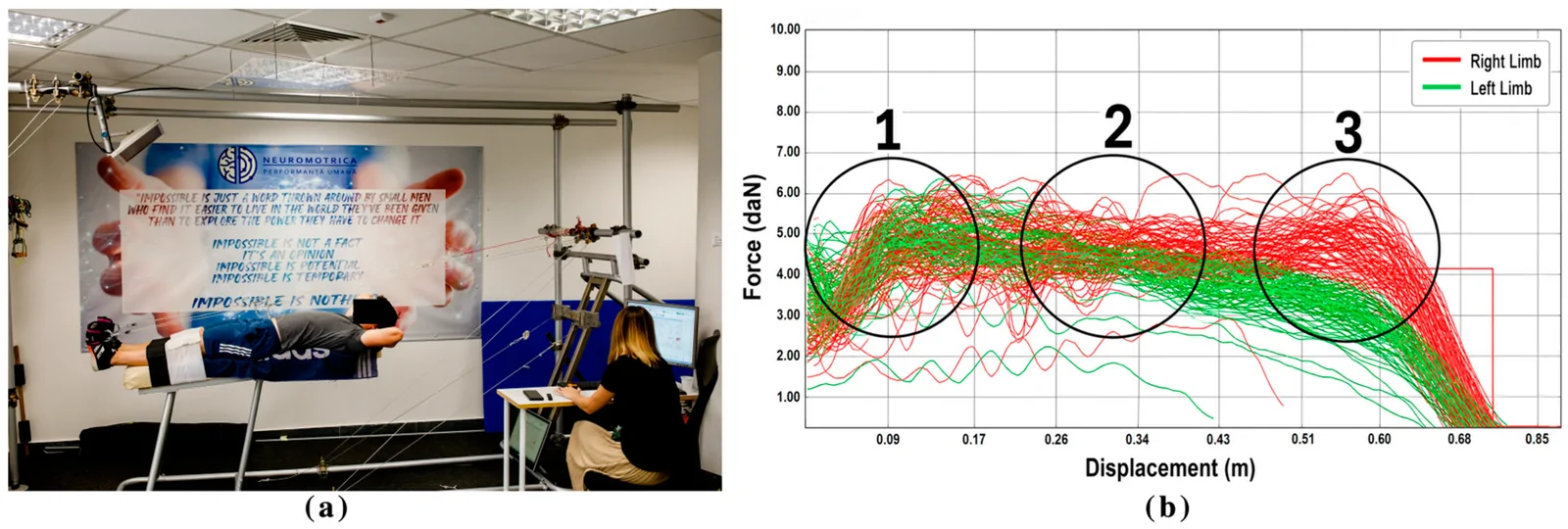
(**a**) Athlete performing a lower-limb extension exercise on the ERGOSIM Condition Simulation Device, with the operator monitoring real-time force output via the CASINOR feedback interface; (**b**) Real-time feedback visualization of a completed Ex3 (knee joint and thigh muscles exercise) from Tr2 (bilateral execution), with force (daN) plotted against position (m), performed at a 4 daN control load, 90% simulator brake intensity, and 0 m/s motor-driven speed, resulting in an average execution speed of 0.29 m/s. Red lines represent the right limb and green lines the left limb (75 repetitions each). Three distinct movement phases are highlighted: delayed bilateral activation at movement onset (1), variable force control during the plateau phase (2), and pronounced inter-limb asymmetry at movement completion (3), characterized by excessive force production in the right limb and insufficient explosive output in the left limb.

Throughout the intervention, athletes received continuous real-time visual feedback on force output and movement timing via the ERGOSIM interface (Figure 1a). Figure 1b illustrates a representative feedback trace for Ex3 during session Tr2, where force (daN) is plotted against position (m); the three characteristic movement phases observed are detailed in the figure caption. Athletes were instructed to reproduce the reference model as accurately and consistently as possible across repetitions.

### 2.5. Testing Protocol

Vertical jump performance was evaluated using the 15 s repeated jump test on OptoJump Next System (Microgate, Bolzano, Italy) [73] in both bilateral and unilateral conditions. This test emphasizes elastic energy return and neuromuscular efficiency by minimizing ground contact time while maximizing flight time [74,75]. Performance was assessed at three measurement points: pre-intervention (T1), post-intervention (T2), and follow-up (T3, seven days later).

All OptoJump assessments were performed by the same experienced investigator following an identical standardized protocol across all three sessions. Data extraction and computation of the MGM-15 outcome variables were performed by a second researcher who was not involved in the intervention delivery and was blinded to the testing time-point codes during data analysis.

Previous studies have demonstrated that the OptoJump system provides excellent validity and high test–retest reliability for the assessment of vertical jump performance under standardized testing conditions [76,77,78]. In the present study, the same device configuration and the same 15 s repeated jump protocol were used across all testing sessions to minimize additional sources of measurement error. To further characterize the repeatability of the present protocol, test–retest reliability across the three testing sessions was evaluated using Cronbach’s alpha, intraclass correlation coefficients (ICC; two-way random-effects model, absolute agreement, average measures), and coefficients of variation (CV). The resulting reliability statistics are presented in Table 4.

### 2.6. Outcome Measures

Raw data were analyzed using the MGM-15 method, an adaptation of the Miron Georgescu test developed by Pierre Joseph de Hillerin, which assesses performance through mean average power (PU), jump height (H), and contact time (CT), collectively capturing neuromuscular efficiency and movement coordination [74,75,79]. Specifically, mean jump height (H) reflects lower-limb explosive output, mean average power (PU) captures coordinated force production per jump, and contact time (CT) represents reactive neuromuscular efficiency. Performance outcomes were computed for both bilateral and unilateral conditions. Bilateral indices included mean jump height (A H_B), mean average power (A PU_B), and ground contact time (A CT_B). Unilateral indices were derived separately for the right limb (A H_R, A PU_R, A CT_R) and the left limb (A H_L, A PU_L, A CT_L). The MGM-15 framework was selected because it integrates jump height, mean average power, and contact time into a single 15s repeated-jump protocol, capturing neuromuscular efficiency under conditions that resemble basketball-specific stretch-shortening demands. Although alternative approaches such as Bosco-type formulas or force plate measurements are widely used, MGM-15 has been previously applied with OptoJump-based repeated jump tests in athletic populations [74,75,79] and provides percentage changes in height and contact time that remain comparable in magnitude to those reported by more conventional methods.

### 2.7. Statistical Analyses

Test–retest reliability across the three testing sessions was assessed using Cronbach’s alpha, the intraclass correlation coefficient (ICC) from a two-way random-effects model with absolute agreement for average measures, and the coefficient of variation (CV). ICC values were interpreted as poor (<0.50), moderate (0.50–0.75), good (0.75–0.90), and excellent (>0.90) reliability [80]. Data are presented as mean ± standard deviation (SD). Normality was assessed using the Shapiro–Wilk test, suitable for the present sample size (n = 21). Sphericity was evaluated via Mauchly’s test; where violated, the Greenhouse–Geisser correction was applied. Repeated measures ANOVA was used to examine time effects for each dependent variable: Jump Height (H), Average Power (PU), and Contact Time (CT) across the three testing sessions (T1, T2, T3), for both bilateral and unilateral conditions. Significant main effects were followed by Bonferroni-corrected pairwise comparisons. Effect sizes are reported as partial eta-squared (η^2^_p_), interpreted according to Cohen’s conventions: 0.01 = small, 0.06 = medium, 0.14 = large [81]. All analyses were performed using IBM SPSS Statistics (version 31.0, IBM Corp., Armonk, NY, USA), with the significance level set at *p* < 0.05. Given this sample size and a repeated-measures ANOVA framework, the design has adequate sensitivity to detect moderate-to-large time effects but is underpowered for small effects. Non-significant results, particularly for CT_L, should therefore be interpreted with a possible Type II error in mind.

## 3. Results

The 15 s repeated jump protocol demonstrated good-to-excellent relative reliability across the three testing sessions (Table 4). Average-measures ICC values ranged from 0.767 to 0.879, while Cronbach’s α ranged from 0.819 to 0.907. All ICC values were statistically significant (*p* < 0.001). Mean coefficients of variation ranged from 13.66% to 24.87%, reflecting the expected biological variability associated with repeated maximal jumping rather than measurement error alone.

Detailed descriptive statistics and percentage changes for all performance variables, calculated via the MGM-15 method, are presented in Table 5. Repeated measures ANOVA results, including F-statistics, effect sizes, and Bonferroni post-hoc comparisons, are summarized in Table 6.

### 3.1. Bilateral Jump Performance

Bilateral jump performance showed clear improvements across the three sessions. Mean bilateral jump height increased significantly over time (F (2,40) = 16.593, *p* < 0.001, η^2^_p_ = 0.453), corresponding to a substantial gain from T1 to T3 (+17.00%; Table 4). Post-hoc analyses indicated that this enhancement was already evident immediately after the intervention (T2) and continued to improve at follow-up, with all time-point comparisons reaching significance (T1–T2, T1–T3, T2–T3; Table 5).

Average power output also increased significantly (F (2,40) = 19.281, *p* < 0.001, η^2^_p_ = 0.491), with consistent gains across both the immediate and short-term follow-up periods. These findings suggest that athletes not only jumped higher but also produced more coordinated force per jump, reflecting enhanced neuromuscular efficiency in bilateral conditions.

Ground contact time decreased significantly over time (F (2,40) = 6.009, *p* = 0.005, η^2^_p_ = 0.231), with a meaningful reduction from pre- to post-intervention and follow-up. This pattern indicates that the intervention enabled athletes to achieve higher jumps with shorter ground contact times, consistent with improved reactive control. Mauchly’s test confirmed sphericities for all bilateral variables (all *p* > 0.05).

**Figure 2:**
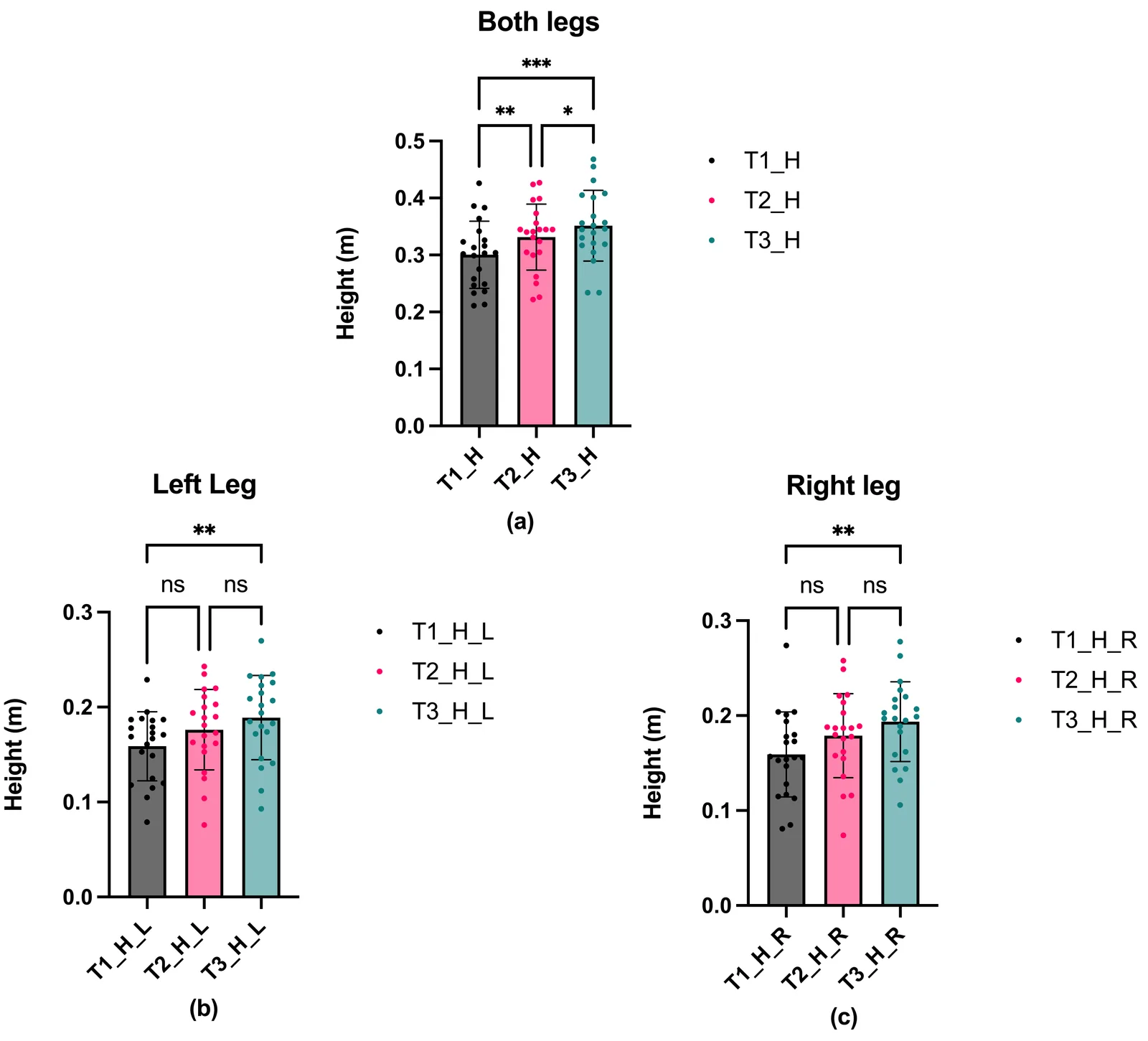
Mean jump height (H) across testing sessions: (**a**) bilateral jumps; (**b**) unilateral jumps—left leg; (**c**) unilateral jumps—right leg. Error bars represent ± standard deviation. Individual data points are shown as jittered dots. ** *p* < 0.01; *** *p* < 0.001; * *p* < 0.05; ns = non-significant (Bonferroni-corrected post-hoc comparisons).

These bilateral adaptations in jump height, average power, and contact time are illustrated in Figure 2, which displays group means and individual data points across T1, T2, and T3.

### 3.2. Unilateral Jump Performance

In unilateral conditions, both limbs exhibited performance improvements, with slightly larger gains on the right side. For the right limb, significant temporal effects were observed for jump height (H_R: F(2,40) = 9.468, *p* < 0.001, η^2^_p_ = 0.321; +22.00%) and average power (PU_R: F(1.433, 28.665) = 11.101, *p* < 0.001, η^2^_p_ = 0.357; +20.20%), with post-hoc tests showing robust T1–T3 differences and an additional late improvement in PU_R between T2 and T3 (Table 5). Together, these changes reflect a sustained enhancement in explosive output and power coordination for the right limb.

Right-limb contact time also decreased over the study period (CT_R: F (2,40) = 4.490, *p* = 0.017, η^2^_p_ = 0.183; −8.30%), with a borderline significant reduction between T1 and T3 (*p* = 0.050). This suggests a modest improvement in reactive efficiency for the right limb, although the magnitude of change is smaller than that observed for bilateral jumps.

For the left limb, significant effects were found for jump height (H_L: F(2,40) = 7.980, *p* = 0.001, η^2^_p_ = 0.285; +18.90%) and average power (PU_L: F (2,40) = 9.370, *p* < 0.001, η^2^_p_ = 0.319; +16.10%), with post-hoc analyses indicating clear T1–T3 improvements. These results show that the intervention also enhanced explosive output and power production on the left side, although the absolute gains were slightly smaller than those observed for the right limb.

No significant temporal effect was observed for left-limb contact time (CT_L: F (2,40) = 2.518, *p* = 0.093, η^2^_p_ = 0.112), despite a 5.0% reduction across the study period (Table 4). This non-significant change should be interpreted cautiously, in line with the limited power of the present design to detect small effects and the potential risk of Type II error for CT_L.

**Figure 3:**
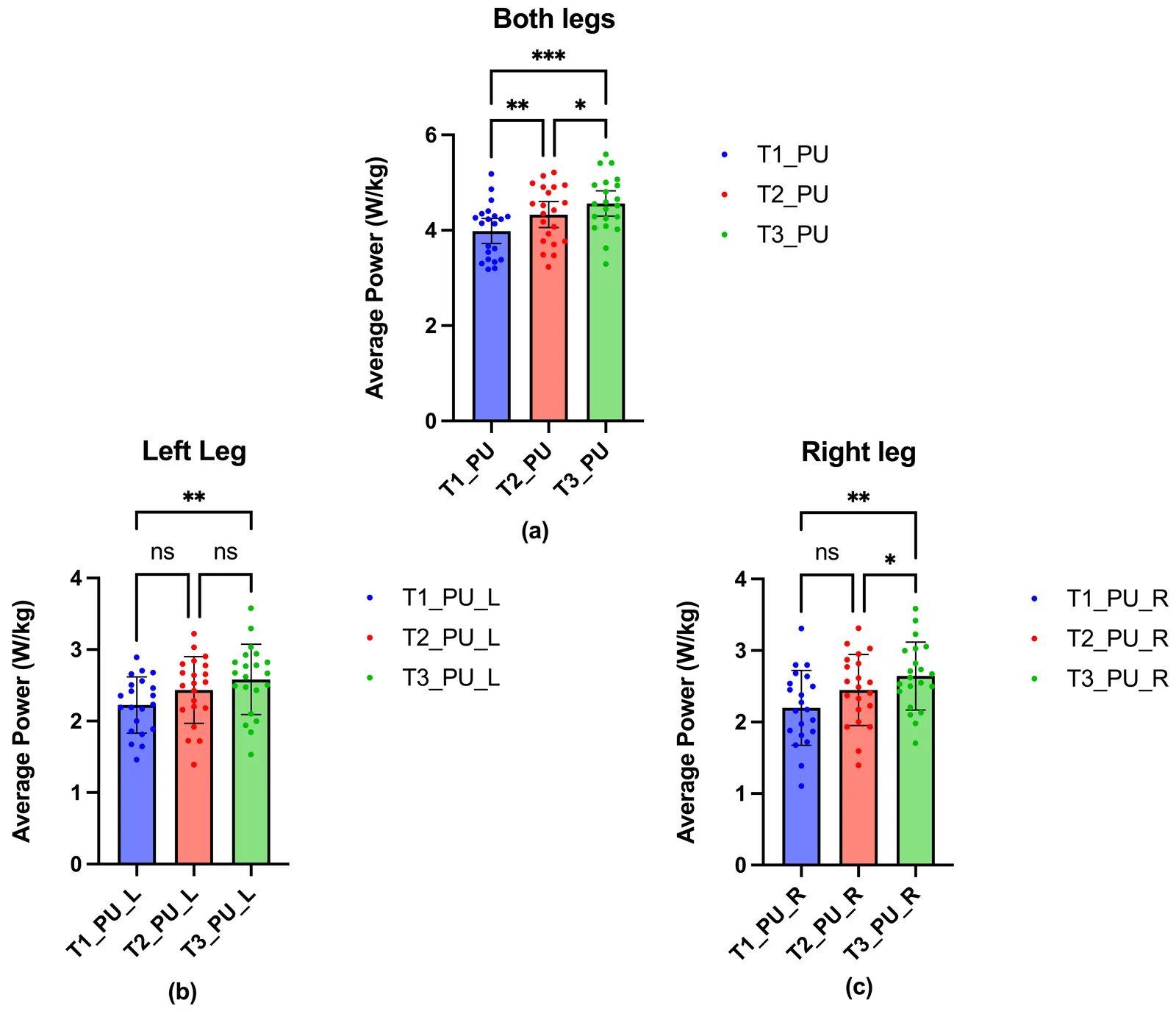
Mean average power output (PU) across testing sessions: (**a**) bilateral jumps; (**b**) unilateral jumps—left leg; (**c**) unilateral jumps—right leg. Error bars represent ± standard deviation. Individual data points are shown as jittered dots. * *p* < 0.05; ** *p* < 0.01; *** *p* < 0.001; ns = non-significant (Bonferroni-corrected post-hoc comparisons).

**Figure 4:**
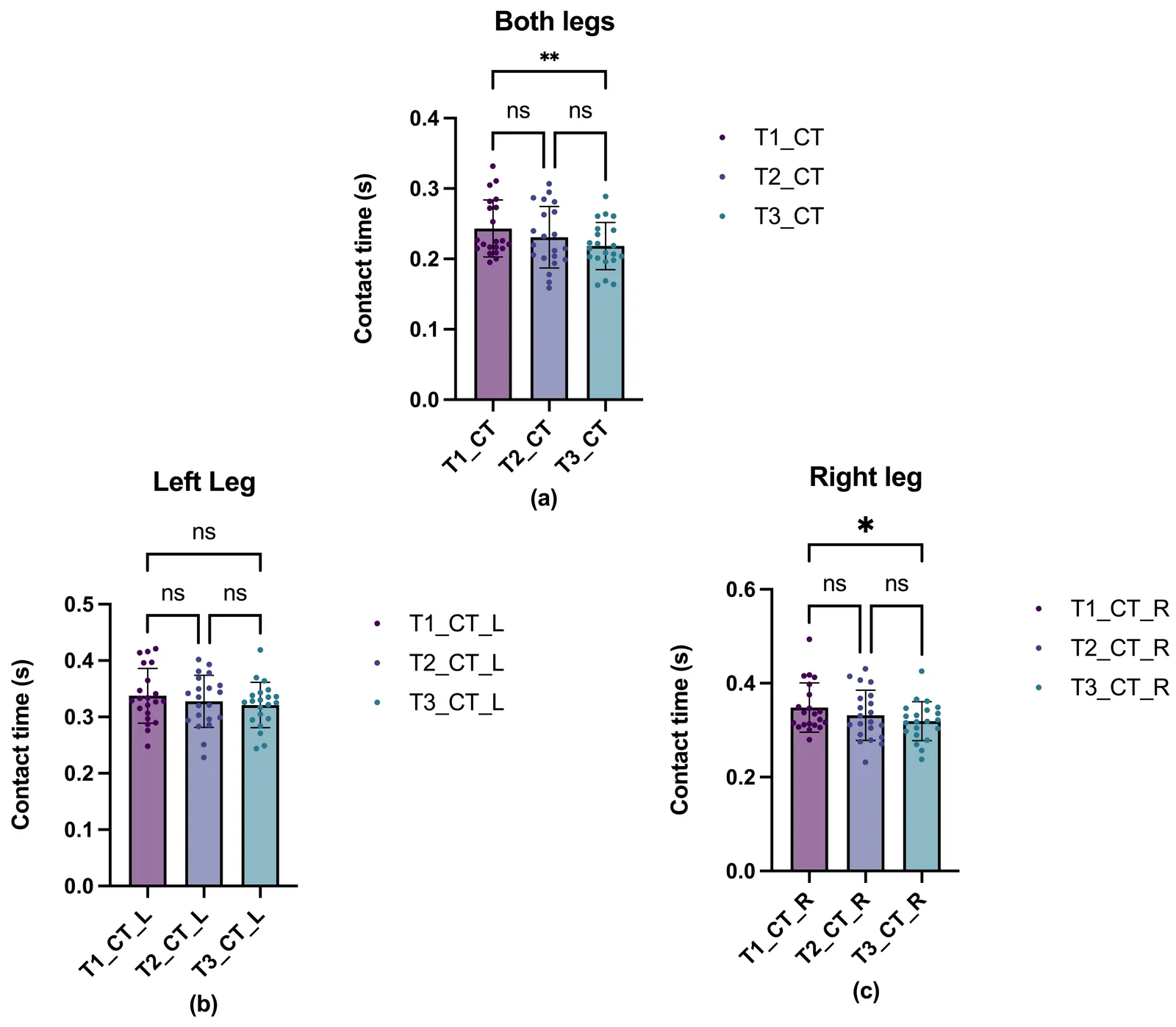
Mean ground contact time (CT) across testing sessions: (**a**) bilateral jumps; (**b**) unilateral jumps—left leg; (**c**) unilateral jumps—right leg. Error bars represent ± standard deviation. Individual data points are shown as jittered dots. * *p* < 0.05; ** *p* < 0.01; ns = non-significant (Bonferroni-corrected post-hoc comparisons).

Figure 3 depicts the unilateral changes in average power output for bilateral and single-leg jumps, highlighting the parallel gains for both limbs and the slightly larger improvements on the right side. Complementary patterns for contact time are shown in Figure 4.

### 3.3. Inter-Limb Symmetry

At baseline (T1), both limbs recorded identical mean jump heights (0.159 m). By T3, values converged to 0.194 m (right) and 0.189 m (left) (Table 4), indicating a trend toward enhanced inter-limb symmetry in explosive output following the intervention. This convergence was mirrored in average power, where both limbs showed comparable percentage increases over time.

From a practical perspective, these patterns suggest that the neuromuscular training protocol promoted more balanced bilateral and unilateral performance, which is desirable in youth basketball players with highly competitive demands. However, contact time adaptations remained more pronounced on the right side, with a significant reduction in CT_R contrasted by a small, non-significant decrease in CT_L. This partly asymmetric evolution of reactive efficiency indicates that, despite global improvements, some inter-limb differences persisted at the level of ground contact dynamics and should be monitored in future work.

## 4. Discussion

The present study investigated the acute and short-term effects of a five-day real-time feedback-based neuromuscular training program on vertical jump performance and reactive efficiency in youth basketball athletes. Significant time effects were observed across all primary bilateral indices and most unilateral measures, with large effect sizes (η^2^_p_ ranging from 0.231 to 0.491) and consistent post-hoc differentiation between assessment points. Notably, performance gains were not only evident immediately after the intervention (T2) but were largely retained at the one-week follow-up (T3), which suggests that the observed improvements were retained over the short term rather than representing only a transient performance fluctuation.

The interpretation of these longitudinal changes is strengthened by the good-to-excellent reliability of the 15s repeated-jump protocol (MGM-15) observed in the present study (ICC = 0.767–0.879). Although the coefficients of variation indicate the expected biological variability associated with repeated maximal jumping, the overall reliability profile supports the interpretation that the observed performance improvements are unlikely to be attributable solely to measurement variability.

### 4.1. Bilateral and Unilateral Adaptations in the Context of Previous Research

The bilateral jump height improvement of +17.0% observed in the present study exceeds the typical gains reported in conventional plyometric interventions of comparable or longer duration. Systematic reviews and meta-analyses consistently report bilateral jump height improvements of 4–15% following structured plyometric programs of 6–12 weeks [6,15,21]. These findings are corroborated by controlled evidence in youth and basketball-specific populations, including adolescent female players, meta-analytic data specific to basketball athletes, and youth-focused programs [14,23,29,30]. Within the constraints of the present quasi-experimental design, these findings suggest that ERGOSIM-based training has the potential to accelerate neuromuscular adaptation. Notably, the observed CMJ and reactive strength gains appear comparable to improvements reported after 4–8 weeks of plyometric training, such as CMJ gains of +7.50–8.20% [34] and reactive strength improvements of up to +19.50% [17], yet were achieved within only five sessions. Even so, the lack of a control or comparison group means that these between-study comparisons cannot be treated as definitive evidence of a causal effect. The rapid improvements observed in the present study are consistent with established principles of augmented feedback and motor learning. Schmidt and Lee [68] describe real-time feedback as a primary catalyst for rapid motor schema formation, enabling athletes to iteratively refine movement patterns within and between repetitions. Sigrist et al. [67] further demonstrated that multimodal augmented feedback, including visual components analogous to those employed in the present protocol, produces superior motor learning outcomes compared to unimodal or delayed feedback conditions. More recently, He et al. [45] demonstrated that real-time visual feedback delivered through augmented interfaces enhances motor learning outcomes and intrinsic motivation in youth team sport athletes, suggesting that the effectiveness of feedback-driven training extends across diverse technological implementations, including the simulator-based approach employed in the present study. These findings are further contextualized by the rapid proliferation of intelligent sensor technologies in sports science, underscoring a broader paradigm shift toward data-driven, feedback-integrated training methodologies [42]. The ERGOSIM’s CASINOR principle may facilitate these mechanisms by integrating the athlete into a closed-loop information system, enabling instantaneous adjustment of force output and movement timing [69,70]. Unlike traditional plyometric programs that prioritize maximal mechanical output through progressive overload [6,17], the present intervention was designed to prioritize neuromuscular precision under low-resistance, speed-controlled conditions, targeting the quality of force application rather than its absolute magnitude.

The significant reduction in bilateral contact time (CT_B: −10.30%, *p* = 0.005, η^2^_p_ = 0.231) further distinguishes this protocol from conventional approaches. Reductions in contact time are widely recognized as a proxy for stretch-shortening cycle (SSC) optimization and reactive neuromuscular efficiency [4,6]. The concurrent improvement in both jump height and power output alongside reduced contact time is consistent with athletes achieving greater vertical displacement within a shorter ground-contact phase, a characteristic commonly associated with improved neuromuscular efficiency and reactive strength development [15,20]. That this pattern emerged after only five sessions may underscore the sensitivity of the SSC to feedback-driven motor learning cues, although this interpretation should again be viewed considering the uncontrolled study design and potential influence of concurrent in-season training.

Unilateral performance mirrored bilateral trends across both limbs, with statistically significant improvements in jump height and average power for both the right limb (H_R: +22.00%, η^2^_p_ = 0.321; PU_R: +20.20%, η^2^_p_ = 0.357) and left limb (H_L: +18.90%, η^2^_p_ = 0.285; PU_L: +16.10%, η^2^_p_ = 0.319). These magnitudes are consistent with findings from studies employing unilateral plyometric programs targeting inter-limb asymmetry correction in youth basketball players [35] and extend them to a feedback-based training context. Notably, the right limb demonstrated a significant reduction in contact time (CT_R: −8.30%, *p* = 0.017), whereas the equivalent reduction in the left limb (−5.0%) did not reach statistical significance (*p* = 0.093), suggesting limb-specific variability in reactive adaptation that warrants further investigation.

This differential limb response may reflect an inherent laterality effect, whereby the dominant limb (right, in most of this cohort) demonstrates greater responsiveness to speed-controlled feedback stimuli. However, the mechanisms underlying this asymmetry cannot be inferred directly from the present findings and remain to be elucidated. Alternatively, the non-significant reduction in CT_L (−5.0%) may represent a floor effect, given that the left limb’s baseline contact time was already marginally lower than the right (0.338 s vs. 0.348 s at T1), leaving less room for a statistically detectable reduction within this sample size. The observed effect size (η^2^_p_ = 0.112), which approaches the threshold for a medium effect, suggests that with a larger sample, the reduction may have reached significance and warrants targeted investigation in future studies. This interpretation is consistent with the post hoc indication that the current design has limited power for detecting small effects, particularly for CT_L.

A particularly noteworthy observation was the tendency toward greater inter-limb symmetry across the intervention period. At baseline (T1), both limbs recorded identical mean jump heights (0.159 m), yet subtle asymmetries in power output and contact time were present. By T3, right and left limb jump heights converged to 0.194 m and 0.189 m, respectively, with consistent patterns across average power and contact time indices (Table 4). The narrowing of individual data point distributions in Figure 2, Figure 3 and Figure 4 may reflect a tendency toward greater performance homogeneity across participants, with lower-performing athletes appearing to demonstrate proportionally larger improvements over time. This finding aligns with previous work demonstrating that unilateral training with real-time feedback promotes bilateral symmetry by enabling athletes to detect and correct latent control asymmetries [55,63,65]. These findings are also consistent with recent evidence suggesting that combined unilateral and bilateral training modalities produce differential adaptation patterns in youth male basketball players [9], a pattern broadly aligned with the limb-specific variability and apparent symmetry convergence observed in the present study.

### 4.2. Practical Implications

The efficiency of the five-day intervention window carries direct implications for applied sports science. The protocol’s brevity suggests it may be integrated as a targeted neuromuscular potentiation microcycle within competitive training blocks, particularly during the pre-competitive taper phase, when minimizing fatigue while preserving neuromuscular sharpness is paramount [37]. The short-term retention of gains at T3 supports feasibility without requiring continuous simulator access. Additionally, the simulator’s real-time monitoring of force output and inter-limb symmetry indicators may position it as a useful tool in Return-to-Play contexts, where objective quantification of lower-limb loading is considered essential for safe progression toward full athletic participation [41,82,83]. The intentionally low applied loads (3–4 daN) suggest this approach complements rather than competes with conventional strength and conditioning programs by refining the technical utility of previously developed force production capacity [4,70]. It should also be acknowledged that training on an unfamiliar, technology-assisted simulator may have introduced a “novelty effect” in this youth cohort, potentially enhancing motivation and engagement independently of specific neuromuscular mechanisms, and this factor should be considered when interpreting the practical implications of the present findings.

### 4.3. Study Limitations and Future Directions

Despite the positive outcomes, several limitations warrant consideration. The sample size (n = 21) and demographic specificity (junior male athletes) constrain generalizability to female athletes, senior populations, or athletes at different developmental stages. A primary limitation is the quasi-experimental, single-group design without a control or comparison group. The absence of a control group prevents the definitive isolation of the intervention effect from potential familiarization influences, regression to the mean, or natural in-season fluctuations in fitness and neuromuscular readiness. Consequently, causal inferences regarding the specific contribution of real-time feedback should be made with caution until confirmed by randomized or controlled trials. However, the magnitude of observed improvements exceeding the typical test–retest variability reported for OptoJump-based assessments [74,75] suggests that familiarization alone is unlikely to fully account for the findings, although this interpretation remains tentative in the absence of formal minimal detectable change thresholds or a control group. At the same time, performance continued to improve at T3, one week after the cessation of the intervention, a trajectory inconsistent with transient familiarization effects, which typically plateau or regress without continued practice [68], and more compatible with short-term neuromuscular consolidation. Nevertheless, this interpretation should be considered within the limitations imposed by the current study design. In addition, concurrent team training during the in-season microcycle was monitored descriptively but not quantified in terms of external or internal load, so its potential contribution to changes between T1–T3 cannot be fully isolated. Furthermore, the long-term retention of these adaptations and their transferability to game-specific performance demands remain to be established. Future studies should include randomized or at least controlled comparison designs (for example, a group performing only standard basketball training), larger and more diverse samples, and additional neuromuscular measures (e.g., EMG or force plates) to clarify mechanisms and transfer to sport-specific tasks such as change-of-direction speed and reactive agility.

## 5. Conclusions

A five-day simulator-based neuromuscular training program incorporating real-time visual feedback was associated with significant, large-effect improvements in bilateral and unilateral vertical jump performance in youth male basketball athletes, with gains retained at the one-week follow-up. The concurrent reduction in ground contact time alongside increases in jump height and power output suggests that reactive neuromuscular efficiency can be meaningfully optimized within an exceptionally short intervention window. Progressive convergence of inter-limb symmetry indices further indicates a corrective effect on neuromuscular coordination extending beyond bilateral performance outcomes, although this finding should be viewed in the context of the absence of a control group.

Within the constraints of this single-group, repeated-measures design, these findings suggest that feedback-driven simulator training may represent a viable neuromuscular potentiation strategy during competitive microcycles and in Return-to-Play contexts. However, the present study does not allow strong causal inferences about accelerated adaptation relative to conventional longer-term plyometric programs, and comparisons with previous work should therefore be considered exploratory. Confirmation through randomized controlled trials, ideally including minimal detectable change thresholds and control or comparison conditions, remains a necessary next step.

## Figures and Tables

**Table 1:** Exercise Descriptions and Biomechanical Characteristics.

Exercise	Target Segment	Starting Position	Movement Description
Ex1	Ankle plantar flexors	Supine on simulator bench; toes pulled toward chest; simulator cables attached to forefoot	Explosive plantar flexion from a dorsiflexed position
Ex2	Hip–knee–ankle triple extension chain	Supine on simulator bench; knees to chest; simulator cables attached to heel plates	Push through heels with leg extension, finishing with explosive plantar flexion
Ex3	Knee extensors (quadriceps)	Seated astride simulator bench; cables attached to dorsal aspect of ankle joint	Forward-upward push of the leg(s) through knee extension

Note. All exercises were performed on the ERGOSIM simulator with real-time force-position visual feedback. Execution mode (unilateral or bilateral) varied across sessions as detailed in Table 3. Low control loads were intentionally selected to prioritize movement control and neuromuscular precision over absolute force production. Exercises were performed across Tr1–Tr5; progression parameters are detailed in Table 2 and Table 3.

**Table 2:** Progression of AS (Tr1–Tr5).

Training No.	Ex1		Ex2		Ex3	
	AS_L [m/s]	AS_R [m/s]	AS_L [m/s]	AS_R [m/s]	AS_L [m/s]	AS_R [m/s]
Tr1	0.06	0.05	0.10	0.10	0.12	0.13
Tr2	0.11	0.11	0.20	0.23	0.27	0.29
Tr3	0.17	0.17	0.40	0.39	0.37	0.37
Tr4	0.30	0.31	0.43	0.44	0.47	0.50
Tr5	0.55	0.51	0.59	0.60	0.55	0.60

Note. AS_L = average speed, left limb; AS_R = average speed, right limb; Ex1 = ankle/calf exercise (3 daN); Ex2 = triple extension exercise (4 daN); Ex3 = knee/thigh exercise (4 daN). Values represent mean speed recorded per session across all repetitions. Progressive increase in AS reflects the reduction in brake intensity from 90% (Tr1–Tr2) to 50% (Tr3) and 30% (Tr4–Tr5).

**Table 3:** Detailed characteristics of the neuromuscular training protocol.

Training No	Execution Type	Exercise	Control Load [daN]	No of Repetitions	Simulator’s Brake Intensity [%]	Simulator’s Motor-Driven Speed [m/s]
Tr1	alternately	Ex1	3	150	90	0.0
		Ex2	4	150	90	0.0
		Ex3	4	75	90	0.0
Tr2	simultaneous	Ex1	3	150	90	0.0
		Ex2	4	150	90	0.0
		Ex3	4	75	90	0.0
Tr3	simultaneous	Ex1	3	150	50	0.0
		Ex2	4	150	50	0.0
		Ex3	4	75	50	0.0
Tr4	simultaneous	Ex1	3	150	30	0.0
		Ex2	4	150	30	0.0
		Ex3	4	75	30	0.0
Tr5	simultaneous	Ex1	3	150	30	0.5
		Ex2	4	150	30	0.5
		Ex3	4	75	30	0.5

Note. Ex1 = ankle joint and calf muscles exercise; Ex2 = triple extension chain exercise; Ex3 = knee joint and thigh muscles exercise. daN = decanewton. Brake intensity (%) indicates the resistance level applied by the electrodynamic brake system, where higher values reflect greater resistance. The reduced repetition count for Ex3 (75 vs. 150 for Ex1 and Ex2) reflects the higher localized muscular demand of isolated knee extension movements. Motor-driven speed of 0.0 m/s indicates no externally imposed baseline velocity; athletes generated movement entirely through voluntary effort. In Tr5, a motor-driven speed of 0.5 m/s was introduced to establish a minimum velocity threshold, requiring athletes to generate force above baseline. Tr1–Tr5 = training sessions 1 through 5.

**Table 4:** Test–retest reliability of MGM-15 variables during the 15-s repeated-jump test across three testing sessions.

Variable	Cronbach’s α	ICC (95% CI)	Mean CV (%)
H_B	0.907	0.848 (0.566–0.942)	18.23
H_L	0.844	0.802 (0.564–0.916)	23.47
H_R	0.849	0.801 (0.546–0.916)	24.87
PU_B	0.891	0.813 (0.464–0.929)	13.72
PU_L	0.845	0.796 (0.537–0.914	18.63
PU_R	0.830	0.767 (0.465–0.903)	20.67
CT_B	0.850	0.821 (0.617–0.923)	16.99
CT_L	0.886	0.879 (0.752–0.947)	13.66
CT_R	0.819	0.795 (0.576–0.910)	14.73

Note. H = jump height (m); PU = average power (W/kg); CT = contact time (s); B = both legs; L = left leg; R = right leg. ICC = intraclass correlation coefficient (average measures, two-way random-effects model, absolute agreement); CI = confidence interval; CV = coefficient of variation calculated as (SD/mean × 100) and reported as the average across the three testing sessions (T1–T3). Cronbach’s α represents internal consistency across repeated measurements. All ICC values were statistically significant (*p* < 0.001).

**Table 5:** Descriptive statistics for bilateral and unilateral performance (N = 21).

Variable	T1 (Mean ± SD)	T2 (Mean ± SD)	T3 (Mean ± SD)	% Change (T1–T3)
Bilateral				
H_B (m)	0.300 ± 0.059	0.332 ± 0.058	0.351 ± 0.062	+17.00%
PU_B (W/kg)	3.983 ± 0.577	4.330 ± 0.600	4.564 ± 0.585	+14.50%
CT_B (s)	0.243 ± 0.041	0.231 ± 0.044	0.218 ± 0.034	−10.30%
Right Limb				
H_R (m)	0.159 ± 0.045	0.179 ± 0.044	0.194 ± 0.042	+22.00%
PU_R (W/kg)	2.198 ± 0.523	2.449 ± 0.500	2.644 ± 0.474	+20.20%
CT_R (s)	0.348 ± 0.053	0.332 ± 0.053	0.319 ± 0.042	−8.30%
Left Limb				
H_L (m)	0.159 ± 0.036	0.176 ± 0.042	0.189 ± 0.044	+18.90%
PU_L (W/kg)	2.225 ± 0.393	2.434 ± 0.467	2.583 ± 0.492	+16.10%
CT_L (s)	0.338 ± 0.049	0.328 ± 0.046	0.321 ± 0.040	−5.00%

Note. H = mean jump height; PU = mean average power; CT = mean ground contact time; B = bilateral; R = right limb; L = left limb. T1 = pre-intervention; T2 = post-intervention (immediately after the five-day protocol); T3 = follow-up (seven days post-intervention). % Change reflects the relative difference between T1 and T3. Values are expressed as mean ± standard deviation (SD). Performance variables were derived using the MGM-15 method. N = 21.

**Table 6:** Repeated measures ANOVA results and Bonferroni post-hoc comparisons.

Variable	F	df	*p*	η^2^_p_	Mean Difference	95% CI of Mean Difference	T1–T2	T1–T3	T2–T3
H_B	16.593	2, 40	<0.001	0.453	−0.0510	−0.077 to −0.024	0.008	<0.001	0.034
PU_B	19.281	2, 40	<0.001	0.491	−0.580	−0.863 to −0.297	0.007	<0.001	0.011
CT_B	6.009	2, 40	0.005	0.231	0.024	0.006 to 0.043	ns	0.008	ns
H_R	9.468	2, 40	<0.001	0.321	−0.034	−0.057 to −0.011	ns	0.003	ns
PU_R	11.101	1.433, 28.665	<0.001	0.357	−0.445	−0.741 to −0.149	ns	0.002	0.011
CT_R	4.490	2, 40	0.017	0.183	0.028	0.004 to 0.057	ns	0.050	ns
H_L	7.980	2, 40	0.001	0.285	−0.030	−0.049 to −0.011	ns	0.002	ns
PU_L	9.370	2, 40	<0.001	0.319	−0.358	−0.583 to −0.133	ns	0.001	ns
CT_L	2.518	2, 40	0.093	0.112	0.0163	−0.000 to 0.037	ns	ns	ns

Note. df = degrees of freedom; η^2^_p_ = partial eta-squared; Mean Difference and corresponding 95% confidence interval (CI) are reported for the primary pre-intervention (T1) versus follow-up (T3) comparison. T1 = pre-intervention; T2 = post-intervention; T3 = follow-up. Sphericity assumed where Mauchly’s test was non-significant: H_B (W = 0.845, *p* = 0.201), PU_B (W = 0.799, *p* = 0.119), CT_B (W = 1.000, *p* = 0.998), H_R (W = 0.766, *p* = 0.080), CT_R (W = 0.862, *p* = 0.244), H_L (W = 0.924, *p* = 0.474), PU_L (W = 0.804, *p* = 0.126), CT_L (W = 0.957, *p* = 0.656). Greenhouse–Geisser correction applied where sphericity was violated: PU_R (W = 0.605, *p* = 0.008). Post-hoc comparisons performed with Bonferroni correction. ns = non-significant (*p* > 0.05).

## Data Availability

Data are contained within the article.

## Abbreviations

The following abbreviations are used in this manuscript:

ANOVA	Analysis of Variance
AS	Average Speed
CASINOR	Computer-Assisted Informational Orthotics
CMJ	Countermovement Jump
CT	Contact Time
daN	decanewton
Ex1, Ex2, Ex3	Specific exercises performed on the ERGOSIM simulator
F	F-statistic
H	Jump Height
MGM-15	Miron Georgescu Method (15-s version)
m/s	meters per second
N	Sample Size
p	Probability value
PU	Average Power Output
SD	Standard Deviation
SSC	Stretch-Shortening Cycle
T1	Pre-intervention measurement
T2	Post-intervention measurement
T3	One-week follow-up measurement
Tr1–Tr5	Training sessions 1 through 5 on the ERGOSIM simulator
W	Mauchly’s test statistic
W/kg	Watts per kilogram
_B	Bilateral execution
_R	Right limb execution
_L	Left limb execution
η^2^_p_	Partial Eta-Squared (Effect Size measure)
